# Easy and rapid method for the determination of gene expression in cumulus cells incubated for oocyte maturation

**DOI:** 10.1186/1477-7827-3-59

**Published:** 2005-10-27

**Authors:** Kanako Kumamoto, Haifeng Wang, Hideaki Yamashiro, Takato Terada

**Affiliations:** 1 Graduate School of Biosphere Science, Hiroshima University, Higashi-Hiroshima, Hiroshima 739-8528, Japan

## Abstract

**Background:**

The objectives of this study were to develop an easy and rapid method for measuring gene expression in a small number of cells by real-time PCR without RNA extraction and purification, and to use this method to determine more precisely IGF-I gene expression in the cumulus cells surrounding oocytes.

**Methods:**

First, after small numbers of cumulus cells were lysed in cell lysis buffer, they were digested with various concentrations of DNase I for different periods at 37°C to determine the optimal conditions for digestion of genomic DNA in the lysate. Since nonspecific amplification was liable to occur when the non-purified RT product of the cell lysate was used for real-time PCR with the given primers, the optimal conditions for Mg2+ and annealing temperature were well investigated. Further, to create the same conditions as in the actual sample reaction for measurement by real-time PCR, RT-minus product was added to the reaction mixture of the standard curve, and then the amplification efficiency was assessed. Next, IGF-I gene expression in cumulus cells collected from cumulus oocyte complexes (COCs) every 4 h during maturation was determined using the developed method.

**Results:**

The optimal conditions for measuring gene expression using the cell lysate from a small number of cells were as follows: incubation of the cell lysate with 0.16 U/microL DNase I with 10 U/microL for 30 min, an Mg concentration of 1.5 mM for amplification of target gene by real-time PCR using RT-product of the cell lysate. When the RT-minus products added to the reaction mixture for the standard curve, which was prepared for purified 18SrRNA plasmid, the PCR efficiency was similar between the sample and the standard.

The IGF-I gene expression in the cumulus cells was elevated up through the first 8 h of the culture and then declined gradually by the end of maturation, with the maximal gene expression (778-fold) seen at 8 h.

**Conclusion:**

It can be concluded that the method developed here, in which equivalent to cumulus cells collected from 0.03–0.075 COCs were employed per reaction, permits rapid and easy determination of target gene expression in a limited number of cells using real-time PCR without RNA extraction.

## Background

*Isobe et al *(2001) have shown that a loss of the gap junction within cumulus cells, i.e. an interruption in the transmission of signaling factors from the cumulus to the oocytes, triggers the resumption of meiosis in pig oocytes [1]. Eppig (2001) also provided biochemical evidence for the signaling factors exchanged between the oocyte and its surrounding cumulus cells, and concluded that the factors are essential for inducing and regulating the differentiation of follicular compartments from one specific developmental stage to the next, thus ensuring the development of the oocyte [2,3]. Furthermore, using specific inhibitors of RNA polymerase II, such as alpha – amanitin or 6-dichloro-1-b-D-ribofuranosyl-benzimidazole, it has been demonstrated that an initial transcriptional event in the cumulus cells that surround the oocyte is an absolute requirement for gonadotrophin-mediated resumption of meiosis in mammalian species including the pig [4], mouse [5] and cow [6,7]. In bovine cumulus-oocyte complexes (COCs), transcriptional events initiated by FSH did not occur when COCs resumed meiosis spontaneously or in the presence of hCG [8]. These findings strongly suggest that gene expressions in the surrounding cumulus cells are essential for the meiotic resumption of oocytes [[9] and [5]].

On the other hand, real-time polymerase chain reaction (PCR) offers substantial advantages over conventional reverse transcriptase – polymerase chain reaction (RT-PCR) for the molecular study of gene expression in any kind of cell, including cumulus cells. Some studies have investigated gene expression by means of real-time PCR using a large number of cumulus cells [10,11] collected from many COCs, since it is difficult to develop a template for investigating gene expression. Only a few gene expressions have been revealed in cumulus cells during oocyte maturation due to the very small number of cumulus cells surrounding an oocyte.

The objective of the present study was to develop a rapid and easy method for determining gene expression using small quantities of the target template in a small number of cells, such as cumulus cells surrounding an oocyte. This method was then used for analyzing IGF-I gene expression in the surrounding cumulus cells during in vitro maturation of bovine oocytes.

## Materials and methods

### Oocyte recovery

The ovaries were obtained at a slaughter house and transported to the laboratory in a saline solution at around 30°C. Oocytes were aspirated from follicles of 2 to 5 mm in diameter using a 10 ml syringe attached to a 21 gage needle. The recovered cumulus-oocyte complexes (COCs) were washed with phosphate-buffered saline (PBS) containing 0.05% polyvinylpyrrolidone (Sigma, USA) and 100 μg/ml kanamycin. The COCs, which were completely surrounded by at least 4–5 layers of non-expanded cumulus in combination with a homogeneous cytoplasm, were used for cumulus cell collection before and after incubation.

### Preparation of the cumulus cell lysates

Three freshly isolated or incubated COCs were vortexed for 2 min in PBS supplemented with 0.002% hyaluronidase, and the oocytes were removed from the cumulus cell suspension. The cumulus cells were washed three times with the PBS provided in a cells-to-cDNA II kit (Ambion, Texas, USA) by centrifugation for 3 min at 4°C at 1200 g, then the pelleted cumulus cells were flash frozen in liquid nitrogen and stored at -80°C until use.

To make a cell lysate, 50 μl of the cell lysis buffer that was provided in the cells-to-cDNA II kit was added to 0.5 ml of small tube (AB gene, Surrey, UK) containing the pelleted cumulus cells and vortexed for 5 min in order to mix the cells. To lyse the cells, the suspension was heated using a block-type thermal cycler system according to the manufacturer's instructions.

### DNase digestion and reverse transcript

Cell lysates were supplemented with the different concentrations (final concentrations of 0.04 U/μl and 0.08 U/μl) of DNase I provided in the cells-to-cDNA II kit or with two concentrations (final concentrations of 0.08 U/μl and 0.16 U/μl) of RNase-free certified DNase I with 10 U/μl (Roche, Penzberg, Germany). They were then incubated at 37°C for various periods. Immediately after the end of the incubation, each tube was heated for 15 min at 75°C to inactivate the DNase.

To synthesize the first strand of cDNA as per the cells-to-cDNA II kit protocol, 5 μl cell lysate, 4 μl dNTP Mix, 2 μl oligo dT and 5 μl RNase-free water were assembled in an RNase-free 0.5 ml tube, then heated for 3 min at 70°C. After this mixture was cooled on ice, 2 μl 10 × RT buffer, 1 μl M-MLV reverse transcriptase and 1 μl RNase inhibitor were added to the reaction tubes. Reverse transcription was carried out for 30 min at 42°C, followed by incubation at 95°C for 10 min. RT-minus products with all the reaction components except for the reverse transcriptase were produced for each sample, and were then employed for real-time PCR in order to demonstrate that the template for the PCR product was cDNA, not genomic DNA.

### Real-time PCR

The primers for 18S rRNA and insulin-like growth factor I (Table 1) were designed using the Gen Bank sequences (accession numbers: AF176811 and X15726) within only one exon, and they had an 80–200 bp product. They were synthesized by Nihon Gene Research Lab Inc. (Sendai, Japan).

**Table 1:** Primer sequences of 18SrRNA and IGF-I for real-time PCR and plasmid construction (†).

Genes	Accession No.	Primer sequences	
18SrRNA	AF176811	5'-AACGGCTACCACATCCAA-3'	5'-GACTCATTCCAATTACAGGGC-3'
		5' – TGACGGGGAATCAGGGTT-3' (†)	5' – CAAAGTAAACGCTTCGGGC-3' (†)
IGF – I	X15726	5'-GGA GTG CAG GAA ACA AGA AC-3'	5'-TTT TGG TAG GTC TTC TGG TG-3'

To construct the standard curve, a DNA fragment amplified with the bovine 18SrRNA primers for plasmid construction was cloned into the PCR 2.1™ vector according to the manufacturer's instructions (Invitrogen Life Technologies, Carlsbad, CA). The plasmid DNA with the target sequence was purified with the help of the QIA Filter Plasmid Maxi Kit (Qiagen, Westburg, The Netherlands). The IGF-I fragments used to generate the standard curve were amplified with the primer for real-time PCR, collected from agarose gel, and then precipitated with ethanol. The concentrations of plasmid and target sequence DNA were evaluated by measuring the absorbance at 260 nm and expressed in a copy number of target fragments per milliliter.

The PCR was performed in a light cycler (Roche, Hercules, CA) using Light Cycler-Fast Start DNA Master SYBR Green I (Roche) as per the kit instructions with the following modifications: the optimal concentration of MgCl2 (1.5 mM) was used for both primers (18SrRNA and IGF-I), which had been explored in experiment 1 and 2 as described below; 200 nM of each primer; 2 μl SYBR Green I master mix; and either 2 or 5 μl of RT product in a final volume of 20 μl.

The basic protocol for real-time PCR was an initial incubation at 95°C for 10 min to activate modified Taq polymerase. Fifty-five cycles of PCR were carried out with denaturation at 95°C for 10 s, then at annealing temperature for 5 s, then extended at 72°C for 10 s. After amplification, a melting curve was generated by heating the samples to 95°C for 0s, followed by cooling to 65°C for 15s and heating to 95°C at a rate of 0.1°C/s at which stage SYBR Green I fluorescence was measured continuously. The melting temperatures and target gene expression were analyzed using Light Cycler Software (version 3.5: Roche). After analysis, the target gene expression was normalized to 18SrRNA.

The RT-minus control from the previous step and minus-template PCR control were included in each reaction of real-time PCR as negative controls. The minus-template PCR should have all the PCR components, with water substituted for the RT reaction aliquot, thus verifying that none of the PCR reagents are contaminated with DNA.

### Experimental design

This study consisted of the two following experimental phases; 1) construction of a rapid and easy method for determining gene expression using RNA crude solution (cell lysate) as a template, and 2) analysis of IGF-I gene expression in cumulus cells using the method developed.

#### Experiment 1: Construction of a rapid and easy method for determining gene expression using RNA crude solution (cell lysate) as a template.

The aim of this experiment was to establish an easy and rapid method for determining gene expression using a small number of cells. The cumulus cells collected from COCs before maturation were lysed in the cell lysis buffer provided in the Cells-to-cDNA II kit. Cell lysate containing genomic DNA was digested for 15 to 60 min with various concentrations of ether the DNase I provided in the Cells-to-cDNA II kit or RNase-free DNase I. Next, the Mg concentration and annealing temperature for real-time PCR using the RT product of the cell lysate were well investigated, since the RT-product may contain the cations that originated in the cell lysis buffer and the RT buffer. Based on the results of this experiment, we determined the optimal conditions for the digestion of genomic DNA in cell lysate, as well as the concentration of Mg and the annealing temperature for real-time PCR when the RT-product of the cell lysate corresponding to the number of cells from 0.075 COC was amplified by real-time PCR with 18SrRNA primers. Further, to ensure that the PCR efficiency (*E *= 10^-1/s ^- 1) was similar between the sample and the standard, we analyzed whether the addition of RT-minus products to the reaction mixture for the standard curve which was prepared for purified 18SrRNA plasmid, affected the PCR efficiency.

#### Experiment 2: Analysis of IGF-I gene expression in cumulus cells using the method developed.

This experiment was designed to test whether IGF-I gene expression in cumulus cells surrounding an oocyte during its maturation can actually be measured by the method developed in Experiment 1. Five COCs were cultured in 100 μl of TCM 199 supplemented with 10% fetal cow serum (FCS; Gibco BRL, NY, USA), 1.3 μg/ml LH (Sigma Chemical Co., St. Louis, MO), 0.6 μg/ml FSH (Sigma), 0.2 mM Na – pyruvate (Sigma), and 50 μg/ml gentamycin in a 96-well plate covered with a plate seal. The oocyte cultivation was carried out at 39°C in a humidified atmosphere of 5% CO_2 _in air. Cumulus cells were collected from COCs cultured for 0 h, 4 h, 8 h, 12 h, 16 h, 20 h, and 24 h, then lysed in the provided cell lysis buffer. The cell lysate was treated for 30 min with 0.16 U/μl of RNase-free DNase I, and then reverse-transcribed with oligo dT primer as described above. The RT-product of the cell lysate was amplified by real-time PCR with IGF-I primers, and the resultant gene expression was normalized to 18SrRNA.

### Statistical analysis

Data are expressed as means ± SD, and statistical comparisons using Stat View were based on an analysis of variance (ANOVA) and on Fisher's PLSD test for post hoc comparisons. A *p*-value < 0.05 was considered significant. A log normal distribution was fitted to each sample set of data, since the logarithmic transformation of real-time PCR data was performed before the statistical analysis.

## Results and Discussion

In investigating gene expression in cumulus cells, RNA extraction from a large number of the cumulus cells collected from many COCs [12] may allow us to determine a low copy-number target. However, it is difficult to determine the kinetics of gene expression in cumulus cells during oocyte maturation, when a large number of cumulus cells from COCs at the same stage could not be collected. Therefore, further studies are needed to develop the method for determining gene expression in a limited number of cells. The crude RNA solution (cell lysate) was used as a template for the reverse transcript.

### Experiment 1: Construction of a rapid and easy method for determining gene expression using RNA crude solution (cell lysate) as a template.

The aim of this experiment was to develop a rapid and easy method for determining gene expression by using minute quantities of target template in a cell lysate prepared from a limited number of cells, such as cumulus cells surrounding an oocyte. The cell lysate contained genomic DNA, and the contaminating genomic DNA might be amplified in real-time PCR, leading to inaccurate quantification of the transcript of interest. In general, the confounding signal from the contaminating genomic DNA could be attenuated by using the primer that was designed to cross an exon/exon boundary and/or by using an RNA extraction method, such as the acid guanidinium thiocyanate-phenol-chloroform method. Besides, complete DNase I digestion of contaminating DNA could be used for real-time PCR. In this study, however, a crude RNA solution (cell lysate) that contained an excess of genomic DNA was subjected to RT as a template, because the present experiment focuses on the construction of a simpler method for determining the gene expression using cell lysate without RNA extraction. Furthermore, the primers employed in this experiment were designed for only single exon in order to develop a method in which even primers made from unknown genetic structures and cloned cDNA could be used. Thus, complete digestion of genomic DNA in the cell lysate was a prerequisite for real-time PCR in the present experiment.

**Figure 1:**
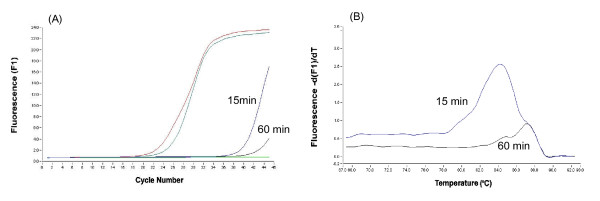
Amplification curves (A) and melting curves (B) of 18SrRNA amplified by real-time PCR using the RT-minus product of the cell lysate treated for 15 min and 60 min with 0.08 U/μl of the DNase I provided in the cells-to-cDNA II kit.

The genomic DNA in cell lysate derived from cumulus cells that were collected from COCs aspirated from 2–7 mm follicles of slaughter house ovaries was digested for 15 to 60 min with 0.04 and 0.08 U/μl DNase I, which was provided in the cells-to-cDNA II kit, and the cell lysate was reverse-transcribed without RT enzyme. The RT-minus product of the lysate was then amplified by real-time PCR with 18SrRNA primers to verify the residual gene contamination (Fig 1). Two amplification curves in late cycles and two melting peaks at 85°C were noted in the data acquired from the amplification of the RT-minus product of the lysate for both periods of incubation with 0.08 U/μl of the provided DNase I. These results showed that the gene contaminants in the cell lysate treated for 15 to 60 min with 0.08 U/μl of the provided DNase I were not digested completely. Likewise, there were two reasonable amplification curves in earlier cycles and two larger melting peaks at 85°C in the data amplified from the RT-minus product of the cell lysate that was treated with the commercial-kit-recommended concentration (0.04 U/μl) of the provided DNase I. This result indicates that the contaminating DNA remained high in the cell lysate after digestion (data not shown).

**Figure 2:**
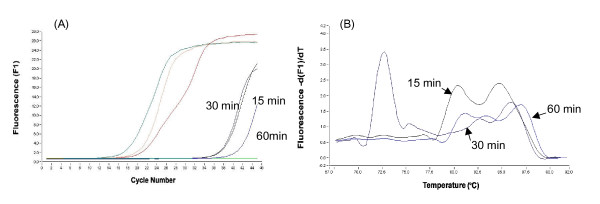
Amplification curves (A) and melting curves (B) of 18SrRNA amplified by real-time PCR using the RT-minus product of the cell lysate treated for 15, 30 and 60 min with 0.08 U/μl RNase-free DNase I.

**Figure 3:**
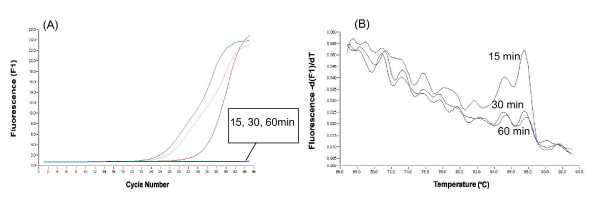
Amplification curves (A) and melting curves (B) of 18SrRNA amplified by real-time PCR using the RT – minus product of the cell lysate treated for 15, 30 and 60 min with 0.16 U/μl RNase-free DNase I.

Further, the cell lysate was treated for 15, 30 and 60 min with 0.08 and 0.16 U/μl RNase-free DNase I. The resultant amplification and melting curve analysis indicated that the cell lysates digested for various time periods with 0.08 U/μl RNase-free DNase I were also remained not to digest genomic DNA contaminants (Fig 2). In contrast, no amplification curve or melting peak of 18SrRNA-specific residual gene amplicons was observed in the RT – minus product of the sample treated for 30 and 60 min with 0.16 U/μl of RNase-free DNase I (Fig 3). Thus, it was concluded that digestion with 0.16 U/μl of RNase- free DNase I for 30 min is enough to eliminate genomic DNA in the celll lysate.

**Figure 4:**
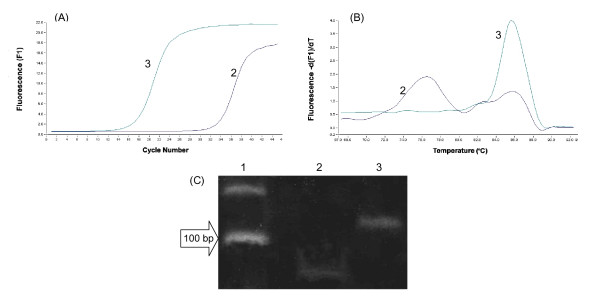
Amplification curves (A) and melting curves (B) of 18SrRNA amplified by real-time PCR using the RT-minus and plus products of the cell lysate (certified residual DNA contamination-free) at 2 mM MgCl_2_, and gel-electrophoresis pattern of PCR product (C). Lane 1: 100-bp Ladder size marker; Lane 2: amplification product of RT-minus (60 bp). Lane 3:. amplification product of RT-plus (120 bp).

The primer pair of the target gene for real-time PCR is usually designed to be the optimal Mg concentration ranging from 2 to 5 mM [13-15]. It is known that the optimal concentration of Mg in real-time PCR is liable to be higher than that in block-type PCR[16]. Thus, determination of the optimal Mg concentration for 18SrRNA within the range of expected Mg concentration (2 mM to 5 mM) was carried out based on the melting and amplification curves of 18SrRNA by real-time PCR using the RT plus and minus products of the cell lysate, which was treated for 30 min with 0.16 U/μl RNase-free DNase I (Fig 4). Amplification curves were found to increase the fluorescence intensity in the product with or without RT when an Mg concentration of 2 mM and an annealing temperature of 60°C were used. Single melting peaks at 85 and 76°C were found in the RT-plus and minus products, respectively. When the PCR products resulting from real-time PCR of the RT-plus and minus products of the lysate were analysed by gel-electrophoresis, single bands of each PCR product were exhibited at 120 bp for the RT-plus product and at 60 bp for the RT-minus product. The real-time PCR of the RT-minus product using an Mg concentration of 3 mM–5 mM at the various annealing temperatures (59°C to 61°C) gave a melting peak at the same melting temperature (76°C) as that given when an Mg concentration of 2 mM was used, indicating that the Mg concentration (3 – 5 mM) for real-time PCR was beyond the optimal Mg concentration for the 18SrRNA primer pair (data not shown).

On the other hand, it has been reported that large molar excess cations, especially Mg2+, produce a nonspecific product [17,18]. Thus, it is possible that the nonspecific bands that were amplified by real-time PCR of the RT-minus product using 2–5 mM Mg concentrations as described above may be produced by a large molar excess of cations that may be contained in the cell lysis buffer and/or RT buffer.

**Figure 5:**
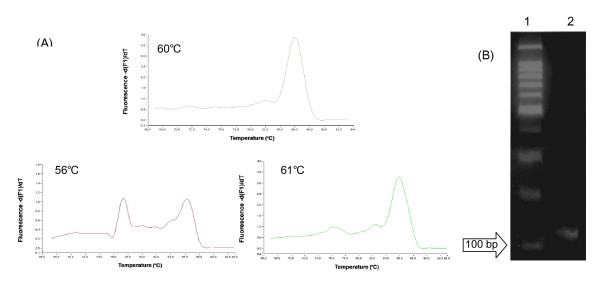
Melting curve (A) amplified at various annealing temparature and gel-electrophoresis pattern (B) of 18SrRNA amplified at 60° of annealing temperature by real-time PCR using the RT product of the cell lysate treated with 0.16 U/μl RNase-free DNase I for 30 min. Lane 1: 100-bp Ladder size marker; Lane 2: amplification product of 18SrRNA (120 bp) in gel-electrophoresis pattern.

The following studies were carried out to optimize the Mg2+ concentration and annealing temperature for the 18SrRNA primer pair of real-time PCR. When an Mg concentration of 1.5 mM and annealing temperature of 56°C were used in real-time PCR, two melting peaks were found at 85 and 77°C, indicating that PCR products amplified at annealing temperature of 56°C contained nonspecific products also (Fig 5). Comparing the specific melting peak for 18SrRNA at 85°C in melting curves amplified at annealing temperature of 60 and 61°C, the peak amplified at annealing temperature of 60°C was larger than that at 61°C. The size of the target gene amplified at 60°C was 120 bp when the size of the target amplicons was certified by 3% agarose gel electrophoresis. These findings showed that an Mg concentration of 1.5 mM, which was slightly low compared with the expected Mg concentration of the primer pair, and an annealing temperature of 60°C were optimal for the determination of 18SrRNA expression using the RT product of the cell lysate.

**Figure 6:**
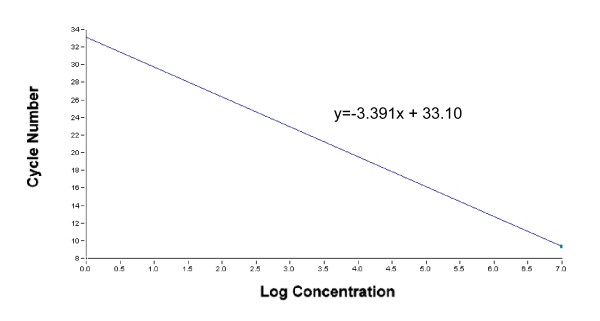
Amplification efficiency and slope of 18SrRNA were assessed by using a reaction mixture of standard curve supplemented with RT-minus control product.

**Figure 7:**
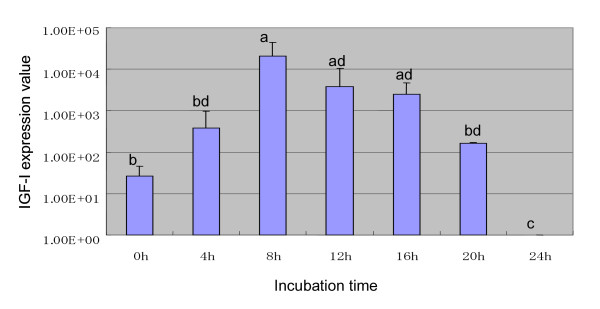
IGF-I expression in cumulus cells surrounding an oocyte during in vitro maturation. Each value represents the mean ± SD. Bars with different letters are significantly different (*P *< 0.05).

However, the PCR efficiency was low in the standard curve of purified plasmid with the sequence of 18SrRNA using an Mg concentration of 1.5 mM (data not shown). This suggests that the 1.5 mM Mg concentration employed for real-time PCR using purified plasmid might be insufficient to produce the same PCR efficiency as was found with the RT product of the cell lysate. Therefore, the RT-minus product of the cell lysate, which was digested with RNase-free DNase I was added to the reaction mixture for the production of the standard curve to ensure an amplification efficiency similar to that observed in the RT product of the cell lysate (Fig 6). The amplification efficiency of the 18SrRNA gene was 0.97 (slope, -3.391), which was similar to the efficiency (0.99) of the standard curve generated from a dilution series of the RT product of the cell lysate (data not shown). Taken together, these findings suggested that the method involving real-time PCR using the cell lysate treated fo 30 min with 0.16 U/μl RNase-free DNase I allowed accurate quantification of gene expression in a small number of the cells.

### Experiment 2: Analysis of IGF-I gene expression in cumulus cells using the method developed.

In this experiment, whether IGF-I gene expression in the cumulus cells surrounding an oocyte during its maturation can actually be measured by the method developed here using cell lysate was tested. An Mg concentration of 1.5 mM and an annealing temperature of 56°C for IGF-I primers proved to produce a high yield of amplicon when assessed in the same way as in Experiment 1. The resultant slope of the standard curve and PCR efficiency were -3.307 and 1.00, respectively.

Transcripts of the target (IGF-I) and reference (18SrRNA) genes were analyzed by real-time PCR using the cell lysate prepared from cultured COCs, and then IGF-I gene expression was normalized by the 18SrRNA gene. Cumulus cells collected from COCs before maturation had low expression ratios of IGF-I genes (Fig 7). IGF-I mRNA expression in the cumulus cells surrounding an oocyte cultured for 8 h was strongly up-regulated, compared to that in cumulus cells collected from COCs before maturation. IGF-I mRNA expression then gradually decreased from 8–24 h of incubation. There was no IGF-I gene expression in the cumulus cells from COCs incubated for 24 h. This is the first report showing that the expression levels of IGF-I in the cumulus cells were dramatically increased during oocyte maturation.

Several growth factors including EGF and IGF-I have been demonstrated to be synthesized in the ovary and to have some effects on cumulus cell functions such as expansion[19] and proliferation [20]. However, Nuttinck et al. recently found that no significant difference was observed in bovine IGF-I gene expression between cumulus cells collected from COCs before maturation and cultured for 24 h, and they then concluded that IGF-I gene expression was unaffected by the differentiation stage of the COCs [21]. In this study, IGF-I mRNA expression in cumulus cells increased abruptly at 8 h of incubation. It is well known that germinal vesicle breakdown (GVBD) occurs in bovine oocytes after 8 h of incubation [22]. This evidence suggests that IGF-I may be responsible for the meiotic resumption of bovine oocytes. This assumption is also supported by several reports showing that IGF-I stimulates the in vitro maturation of cattle [23,24] and rat [25] oocytes, and mediates the meiotic resumption of Xenopus[26].

## Conclusion

In conclusion, since IGF-I gene expression in cumulus cells surrounding an oocyte during its maturation can actually be measured by the method that employs cell lysate derived from a limited number of cells, we can conclude that the method described in this study is a useful tool for the rapid and easy analysis of target gene expression.

## Authors' contributions

KK was responsible for the design, coordination of the experiments, carried out the molecular genetic studies, and drafted the manuscript. HW carried out the oocyte maturation. HY collaborated in the oocyte maturation and molecular genetic studies. TT was responsible for design and coordination of the study. He analyzed the data and helped to drafted the manuscript. All authors read and approved the final manuscript.
